# Human Cystic Echinococcosis in Lebanon: A Retrospective Study and Molecular Epidemiology

**DOI:** 10.1007/s11686-021-00453-w

**Published:** 2021-07-15

**Authors:** Gaelle Joanny, Maria Grazia Cappai, Francesca Nonnis, Claudia Tamponi, Giorgia Dessì, Naunain Mehmood, Julien Dahdah, Chadi Hosri, Antonio Scala, Antonio Varcasia

**Affiliations:** 1 Dipartimento di Medicina Veterinaria, Università degli Studi di Sassari, Sassari, Italy; 2 Department of Zoology, University of Sargodha, Sargodha, Pakistan; 3 School of Medicine, Division of Thoracic Surgery, Department of Surgery, Lebanese American University, Byblos, Lebanon; 4 Faculty of Agronomy and Veterinary Medicine, Lebanese University, Dekwaneh, Lebanon

**Keywords:** Cystic echinococcosis, Zoonosis, *E. granulosus s.s.*, Haplotypes

## Abstract

**Purpose:**

Human cystic echinococcosis (CE) is a zoonotic parasitic disease that constitutes a public health challenge and a socio-economic burden in endemic areas worldwide. No specific surveillance system of CE infections in humans exists in Lebanon. The incidence and trends over time have not been documented. The current study aimed to assess the demographic and epidemiologic features of human CE surgical cases over a 14-year period in the five main regions of Lebanon.

**Methods:**

From 2005 to 2018, a total of 894 surgically confirmed cases of hydatidosis were recorded from five anatomy and pathology laboratories.

**Results:**

The mean annual surgical incidence was 1.23/100,000 inhabitants. Over the span of these years, the incidence increased from 0.53 to 1.94 cases/100,000 inhabitants in 2005 and 2018, respectively. CE is present in Lebanon with an uneven distribution from one region to the other with higher prevalence in Bekaa (29.0%), a rural area where sheep raising is widespread. Human CE cases were more common in females (60.1%) than in males (39.9%) and a high burden of infection was reported for the age group of 30–39 years. Besides, 66.7% of the cases expressed only liver complications whereas, 20.5% showed predilection towards lungs. The 7.8% of cases presented cysts in other organs, and 1.3% showed multiple localizations. Additionally, predominant involvement of *Echinococcus granulosus *sensu stricto was recorded in human infections. Comparison of *Echinococcus granulosus s.s.* populations from different Mediterranean countries also revealed high gene flow among this region and sharing of alleles.

**Conclusion:**

The current study is a step forward to fill the gap of knowledge for the hydatidosis in Lebanon where the lack of epidemiological data and control measures have resulted in higher incidence of human CE.

**Graphic Abstract:**

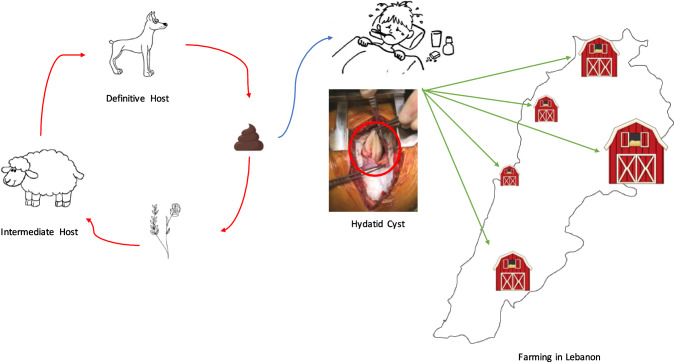

## Introduction

Cystic echinococcosis (CE), or hydatidosis, is one of the most severe parasitological diseases ranking as second most concerning food-borne disease globally [1, 2]. Despite long-known history and considerable scientific advancements, CE still represents a persistent zoonosis with significant socio-economic impacts [1–3]. CE is one of the most important parasitic diseases in the Mediterranean region (MR) and the Middle East [1, 4, 5]. In general, CE is common in pastoral regions where sheep, cattle, and camelids are prominent and is present worldwide with endemic foci on every inhabited continent [1]. Overall economic losses due to this disease are estimated at two billion US$ annually and CE is believed to affect more than one million people worldwide [6].

CE is caused by the tapeworms belonging to the *Echinococcus granulosus *sensu lato (*E. granulosus s.l.*) species complex comprised of ten separate genotypes (G1–10) and *E. felidis*, each with specific geographical distribution and host affinities [6–8]. Its natural transmission involves canids as definitive hosts which harbor the adult tapeworms in intestines and shed eggs with feces which are taken up by the livestock (intermediate hosts) where the larvae grow into a fluid filled hydatid cyst. The definitive hosts, in turn, acquire infection through eating cyst infected organs of the slaughtered animals. Humans are the dead-end (aberrant hosts) in this life cycle acquiring infection through ingestion of infectious eggs while in contact with dogs [9]. Alternatively, indirect transmission can also occur by ingestion of contaminated food, water or soil carrying the eggs of the parasite [3, 10, 11]. The infection starts when oncospheres released from ingested eggs penetrate the intestinal wall, then the larvae migrate through the portal venous system reaching the liver and possibly various other internal organs developing into hydatid cysts (metacestode). As these cysts grow slowly, this first phase of CE occurs asymptomatically [3, 9, 9]. Only later, when cysts reach a considerable size, which can be responsible of organ dysfunction, could symptoms associated with involved organs arise. Additionally, the risk of rupture of cysts could possibly lead to anaphylactic shock and death, or to the dispersion of oncospheres resulting in multiple secondary echinococcosis disease [3, 12]. Human infections have largely been attributed to *E. granulosus *sensu stricto (*s.s.*) (G1 genotype, sheep strain) due to its cosmopolitan distribution and maintenance through sheep–dog cycle [13]; genotype G3 is also implicated in human CE [14]. Other taxa having significant contribution in human CE are genotypes G6 and G7 which usually transmit through camels, goats, and pigs, respectively, in areas where these genotypes have predominant occurrence in animals [13].

The presence of *E. granulosus s.l.* tapeworms as well as the prevalence of CE in both animals and humans has been extensively recorded around the Mediterranean basin, including France, Spain, Italy, Greece, Turkey, Cyprus, Syria, Israel, Egypt, Libya, Tunisia, Algeria, and Morocco and from all the Middle Eastern countries [1, 47]. However, scarce recent information regarding the epidemiology and impact of the different *E. granulosus s.l.* species and genotypes is available for Lebanon [15] despite CE being an important public health problem in the country [16]. Furthermore, all the socio-economic conditions contributing to disease perpetuation are prominently present in Lebanon and account for the endemic nature of hydatidosis in this country. Recently very high rate of infection (62.9%) is reported among sheep in Lebanon with *E. granulosus s.s.* being the causative agent for the majority of infections [17] speculating high risk for human population. For these reasons, an epidemiological survey was carried out to investigate the distribution of CE in Lebanon. Additionally, metacestodes isolated from patients diagnosed with CE in Lebanese hospitals were genetically characterized as to get a better understanding of what *E. granulosus s.l.* species and genotypes are represented in this region of the Mediterranean.

## Materials and Methods

### Survey and Data Collection

**Fig. 1:**
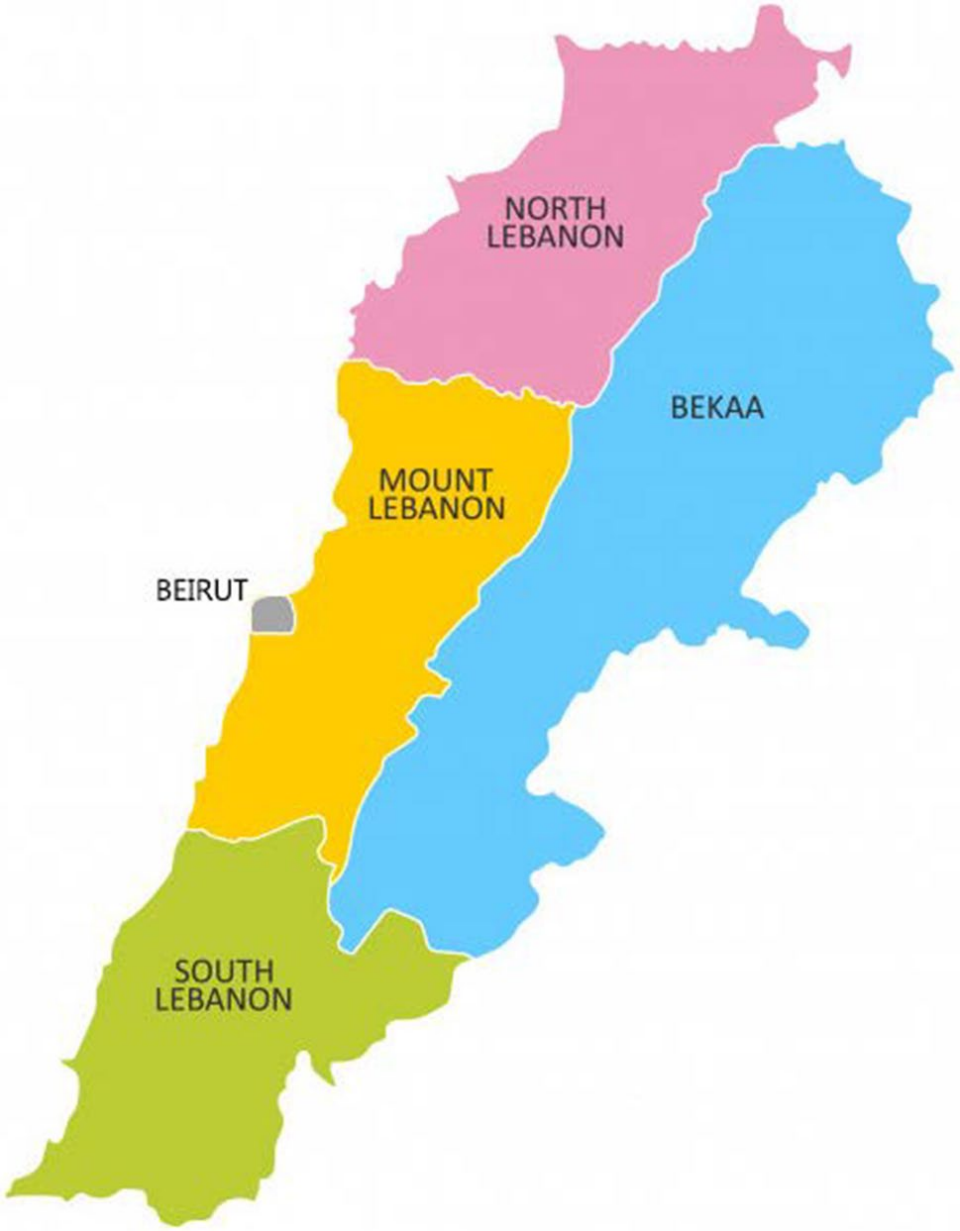
Human CE cases distribution over five Lebanese regions between 2005 and 2018. According to hospitals’ data during this period, it was registered 121 cases of hydatid cysts in North of Lebanon, 222 cases in Mount Lebanon, 132 in Beirut, 160 in South, and 259 in the Bekaa region

In total, data for 894 surgically confirmed cases of human CE were obtained from five main pathology laboratories, each located in one of the five principal Lebanese regions: Beirut, Bekaa, Mount Lebanon, North Lebanon, and South Lebanon (Fig. 1). Each pathology laboratory assists the area hospitals which lack the specialized pathology section and subcontract these services to external specialized units. This puts our selected laboratories as significant representatives of the area dynamics.

Retrospective epidemiological and clinical data from the laboratories included in the investigation were obtained for all patients who underwent surgical treatment after confirmation of CE diagnosis by radiology and histology (microscopic and macroscopic), between January 2005 and December 2018. Only data regarding patients who underwent surgical removal of a hydatid cyst were included in this research.

The collected data consisted of hospital discharge records (HDRs) containing patients’ personal and medical information. Only a selected number of parameters from the HDRs were used during the analyses: gender, age, location of the excised cyst(s), and year of treatment. Finally, the gathered information from each patient was centralized in a spreadsheet using the software Microsoft Office Excel^®^.

Additionally, human CE data were also collected from the register of the Ministry of Health in Lebanon (MOH, available online) [18] to compare that with HDRs.

### Data Analysis and Statistics

All data (i.e., cyst surgical removal site, age and gender of patients, and hospital location) were analyzed with a descriptive analysis, according to region, age, gender of patients and lesion site. Mean annual incidence rates of surgery have been calculated on the basis of the total population in Lebanon from 2005 to 2018 obtained from the World Bank Data and WHO data (https://data.worldbank.org/country/LB). Moreover, the data from the pathological laboratories were compared with the data retrieved from the MOH.

### Molecular Analysis

To identify the etiological agents of the human CE in Lebanon, four freshly excised cysts removed from lungs and liver of four donors were collected, under the supervision of a specialist doctor, from patients admitted to different hospitals located in North and Mount Lebanon regions. The excised germinal layer and the cystic liquid of the hydatids were preserved in ethanol (95%) before being analyzed by the Department of Veterinary Medicine (University of Sassari, Italy). In addition, ten paraffin-embedded fixed samples from surgically confirmed CE cases were collected from the pathology departments of two hospitals located in the Bekaa region.

Thin sections of paraffin-embedded samples were soaked in xylol and with subsequent soaking in absolute ethanol to remove the paraffin prior the DNA extraction. DNA from fresh (*n* = 4) and paraffin-embedded samples (*n* = 4) was extracted using NucleoSpin Tissue (Macherey–Nagel GmbH % Co.KG, Düren, North Rhine-Westphalia, Germany). A fragment within the cytochrome c subunit 1 (*cox1*) was amplified from DNA of both fresh and paraffin-embedded samples using two different set of primers and protocols. In particular, the primers COIF (5′-TTGAATTTGCCACGTTTGAATGC-3′) and COIR (5′-GAACCTAACGACATAACATAATGA 3′) were used to amplify a fragment of approximately 800 bp from DNA of fresh samples, as previously described [19]. Additionally, a fragment of 396 bp was amplified using the primers JB3 (5′-TTTTTTGGGCATCCTGAGGTTTAT-3′) and JB4.5 (5′-TAAAGAAAGAACATAATGAAAATG-3′) from the DNA of the paraffin-embedded samples according to Bowles* et al.* [20], given the DNA fragmentation due to the fixation process of those samples. DNA of the fresh cyst material was also amplified for *nad5* gene (680 bp) for proper discrimination of *E. granulosus s.s.* genotypes [21].

PCR products were purified using a Nucleospin Gel and PCR cleanup (Macherey–Nagel GmbH & Co. KG, Düren, North Rhine-Westphalia, Germany) and sent to an external sequencing service (Eurofins Genomics, Ebersberg, Germany) for bidirectional sequencing. Obtained electropherograms for partial *cox1* regions (800 bp and 396 bp) were aligned with their respective reference sequences [19, 20] for determination of genotypes.

*E. granulosus s.s.* sequences for the human CE isolates (this study) and intermediate hosts from Lebanon [17] and other Mediterranean states (Iraq, Jordan, Algeria, Italy, Spain, Turkey, Tunisia) were compared to understand demographic and transmission patterns for this region. Available mitochondrial *cox1* nucleotide sequences for Iraq (*n* = 38), Jordan (*n* = 12), Algeria (*n* = 81), Italy (*n* = 118), Spain (*n* = 38), Turkey (*n* = 112), and Tunisia (*n* = 83) were retrieved from GenBank database and in total 562 sequences were analyzed. Due to lower number of sequences for France, Greece, Albania, Morocco, and Libya, these Mediterranean countries were not included in the analysis. Dataset for the partial *cox1* gene was trimmed to equal lengths (720 bp) and computed in DnaSP [22] for information on haplotypes and polymorphism. Furthermore, population diversity indices (haplotype and nucleotide diversities), neutrality indices (Tajima’s *D* and Fu’s Fs) and pairwise fixation index (F_st_) were calculated using Arlequin [23]. A haplotype network was generated through PopART software providing information on all haplotype linkages [24].

## Results

The analysis of data provided the temporal and spatial distribution of CE in the patients who underwent the surgical removal of hydatid cysts in different hospitals of Lebanon. Based on HDRs, a total of 894 human CE cases occurred between 2005 and 2018 in five different Lebanese regions (Beirut, Bekaa, Mount Lebanon, North, and South). Retrospective data analysis yielded that CE was present in all regions with the Bekaa region showing significant highest prevalence (29.0%; 259/894) followed by Mount Lebanon (24.8%; 222/894), South Lebanon (17.9%; 160/894), Beirut (14.8%; 132/894), and North Lebanon (13.53%; 121/894) (Fig. 1).

**Fig. 2:**
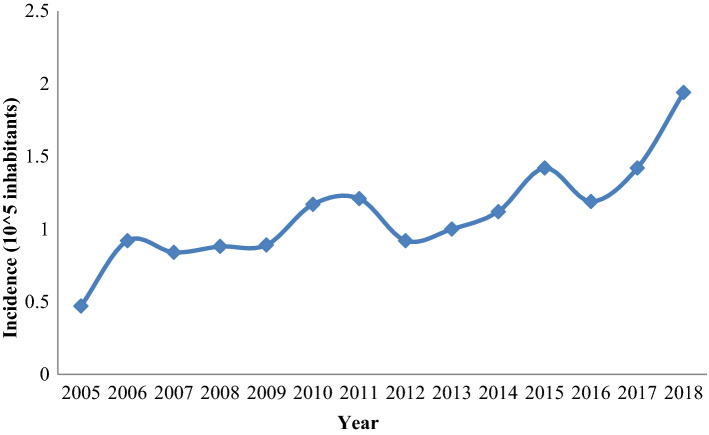
Incidence rates of surgically removed CE in Lebanon per 100,000 inhabitants during 2005 and 2018

The total number of registered surgical cases increased from 21 in 2005 to 133 in 2018 with a mean annual incidence rate of 1.23/100,000 inhabitants. Across the years, an increasing trend could be pointed from the year 2005 (0.53/100,000 inhabitants) to date (1.94/100,000 inhabitants) (Fig. 2).

**Fig. 3:**
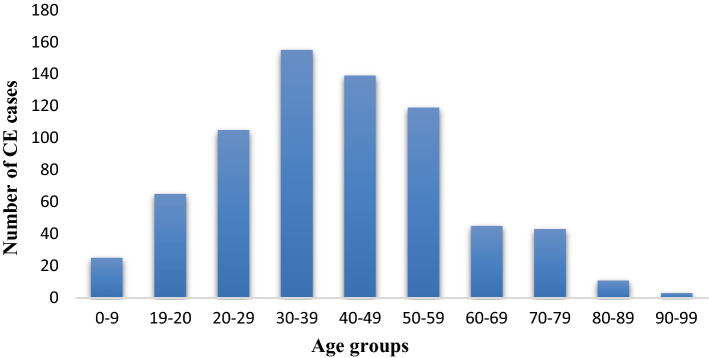
Age distribution of human CE cases identified in Lebanon during 2005 and 2018

Age distribution of the CE cases in Lebanese population showed that patients belonged to multiple age groups ranging between 4 and 94 years (mean age 41). Highest prevalence was observed for the age cohort of 30–39 years (17.3%; 155/894) (Fig. 3). Furthermore, 60.1% (537/894) of recorded cases referred to females and 39.9% (357/894) referred to males.

**Fig. 4:**
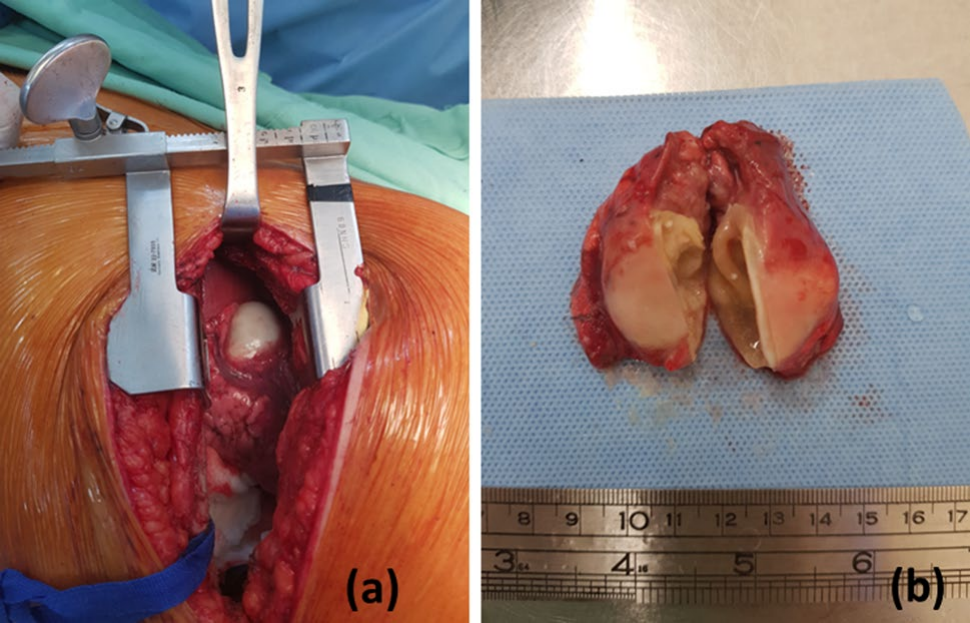
**a** Intraoperative CE found in human lungs in Lebanon. **b** Complete resection of a 4 cm lung hydatid cyst showing the pericyst and the inner germinal layer

Data regarding the cysts localization were available for 861 cases (33 cases were unspecified): 849 CE cases with single organ infection and 12 with multiple organ infections. The majority of patients were affected by liver echinococcosis (66.7%), followed by pulmonary CE (20.5%). The 7.8% of cysts were present in other sites like the abdomen, the spleen, bones, kidneys and ovaries, whereas 1.34% cases showed multiple cyst localization (Table 1, Fig. 4).

**Table 1:** Distribution of CE cases based on their lesion site

Site	Number of cases	Percentage (%)
Liver	597	66.78
Lung	183	20.47
Abdominal cavity	15	1.68
Spleen	17	1.9
Bone	11	1.23
Kidneys	11	1.23
Liver and lung	8	0.89
Pelvis	3	0.34
Liver and spleen	2	0.22
Ovaries	2	0.22
Adrenal gland	1	0.11
Bladder	1	0.11
Brain	1	0.11
Breast	1	0.11
Heart	1	0.11
Inguinal	1	0.11
Intramuscular	1	0.11
Liver and abdomen	1	0.11
Liver, kidneys, and diaphragm	1	0.11
Pancreas	1	0.11
Skin	1	0.11
Spine	1	0.11
Unspecific site	33	3.69

**Fig. 5:**
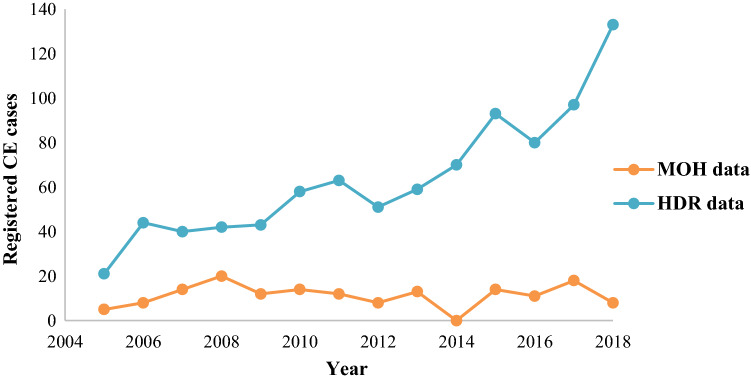
Comparison between CE cases registered per year according to the Ministry of Health (MOH) and the collected data from the five anatomy and pathology hospital laboratories (HDR)

Furthermore, a comparison between our survey and data obtained by the MOH was made to check the similarities in the number of cases of CE reported per year and gender distribution. Although our survey included only surgically confirmed CE cases from a limited number of private, public and university hospitals in Lebanon, we could note that the MOH presented a lack in case reporting and data registration (Fig. 5). In regards to gender of patients, females were more predisposed to CE than males in both data sets collected directly from the hospitals and those recorded by the MOH.

**Fig. 6:**
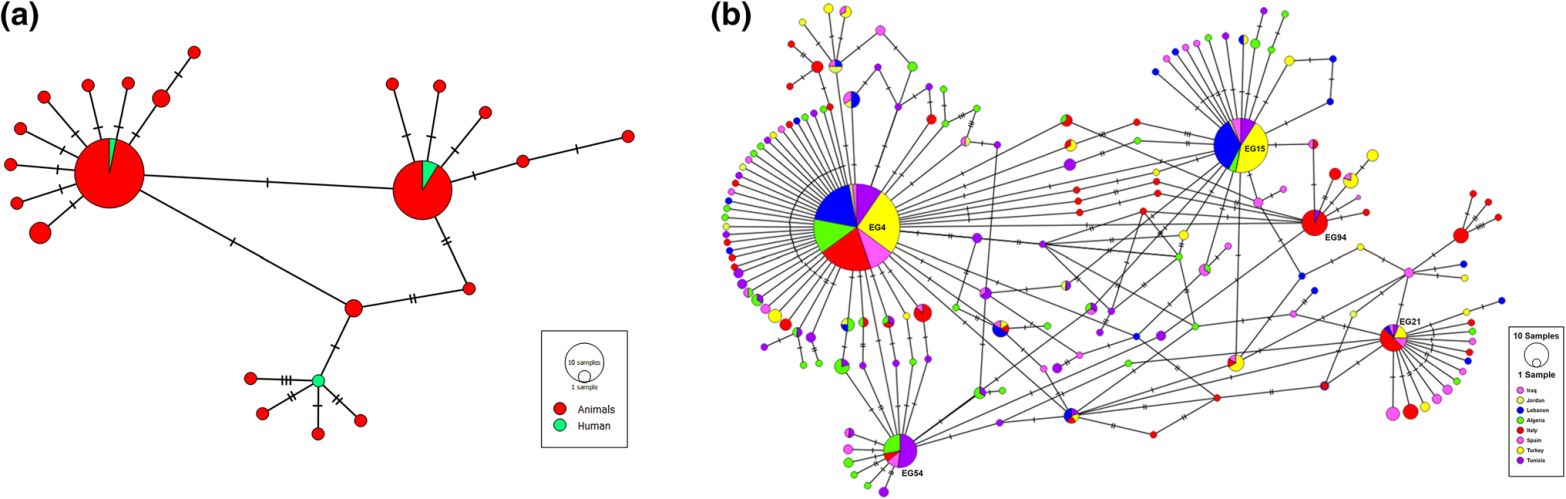
**a** Haplotype network for *E. granulosus s.s.* sequences from the intermediate hosts of Lebanon. **b** Haplotype network for *E. granulosus s.s.* sequences from Lebanon and other Mediterranean states (Iraq, Jordan, Algeria, Italy, Spain, Turkey, Tunisia). Hatchmarks represent number of mutations. Size of the circle indicates its prevalence in the studied populations. Haplotype labels are only kept for dominant haplotypes

Genotyping of the examined isolates revealed the involvement of *E. granulosus s.s.* in the human CE in Lebanon. Sheep strain (G1 genotype) was identified from seven isolates (87.5%) whereas, buffalo strain (G3 genotype) was characterized from a single cyst removed from liver of a female patient. Moreover, four sequences amplified from human isolates for partial *cox1* (800 bp) were compared with the animal isolates obtained from Lebanon showing shared haplotypes between these hosts (Fig. 6a). The population structure analysis encompassing the *E. granulosus s.s.* populations from different Mediterranean countries revealed the presence of 167 haplotypes having 130 point mutations. Predominant occurrence of two haplotypes, EG4 (29.94%) and EG15 (11.74%), followed by EG54 (4.44%) was observed in the haplotype network (Fig. 6b).

Molecular analysis of the *E. granulosus s.s.* sequences from the MR revealed a high haplotype diversity (0.8297 ± 0.0109) and low nucleotide diversity (0.00308 ± 0.00010) (Table 2). Neutrality indices were highly negative (*D* = − 2.5711, Fs = − 373.161) and significant (*p* < 0.01) suggesting the presence of rare haplotypes and, therefore, population expansion. The pairwise fixation index values were also very low ranging from − 0.02051 (Iraq and Jordan) to 0.12014 (Lebanon and Spain) indicating high gene flow among these countries (Table 3).

**Table 2:** Diversity and neutrality indices for *E. granulosus s.s.* populations from Lebanon and other Mediterranean countries

Country	Number of sequences		Diversity indices		Neutrality indices	
		Hn	Hd ± SD	Nd ± SD	Tajima’s *D*	Fu’s Fs
Iraq	38	30	0.9829 ± 0.0114	0.003966 ± 0.002371	− 2.02706*	− 26.3933*
Jordan	12	10	0.9697 ± 0.0443	0.003068 ± 0.002048	− 1.63391*	− 7.42411*
Lebanon	80	23	0.7614 ± 0.0368	0.001877 ± 0.001279	− 2.10582*	− 19.7808*
Algeria	81	43	0.9176 ± 0.0249	0.003261 ± 0.001992	− 2.41293*	− 27.0388*
Italy	118	44	0.8950 ± 0.0217	0.003453 ± 0.002078	− 2.07815*	− 26.8700*
Spain	38	11	0.8065 ± 0.0587	0.002938 ± 0.001859	− 0.58533	− 3.21021*
Turkey	112	23	0.7846 ± 0.0300	0.002306 ± 0.001513	− 1.85399*	− 16.7074*
Tunisia	83	41	0.9342 ± 0.0170	0.003151 ± 0.001937	− 2.28689*	− 27.0323*
Overall	562	167	0.8297 ± 0.0109	0.00308 ± 0.00010	− 2.57110*	− 373.161*

*Significant at *p* < 0.05

**Table 3:** Pairwise fixation index (Fst) for studied populations of *E. granulosus s.s.* from Lebanon and other Mediterranean countries

*Fst*	Iraq	Jordan	Lebanon	Algeria	Italy	Spain	Turkey
Iraq	–						
Jordan	− 0.02051	–					
Lebanon	0.00266	0.00514	–				
Algeria	0.05306*	0.01960	0.06149*	–			
Italy	0.04977*	0.03177	0.07169*	0.06667*	–		
Spain	0.06806*	0.05790	0.12014*	0.06529*	0.03108*	–	
Turkey	0.00534	0.00437	0.00077	0.06608*	0.06057*	0.10726*	–
Tunisia	0.06501*	0.03467*	0.08161*	0.00017	0.07418*	0.08039*	0.08172*

*Significant at *p* < 0.05

## Discussion

This is the first comprehensive retrospective study on human epidemiology of CE in Lebanon to date since a survey carried in 1961 [25]. From previous studies it is well known that CE is highly endemic in Lebanon, with an incidence of 3.8/100,000 inhabitants [26]. However, the incidence seemed to decrease in recent years, although no evidence is currently available in the literature to support this statement [15]. As CE is an important health concern in both humans and animals and is paired with considerable economic burden due to the medical treatment costs, work impairment, morbidity and mortality [27], we decided to undertake this current study and to assess the status of human CE and the population structure of *E. granulosus s.s.* in Lebanon and its neighboring countries. Our aim is to better understand the transmission dynamics of this parasite in this region.

Estimation of regional prevalence revealed the highest frequency of human CE in the Bekaa region which is a fertile valley in eastern Lebanon and the most important farming region of the country. The highest prevalence of CE usually occurs in regions with extensive and traditional sheep farming [28] leading to human infections [13]. Previously, higher CE prevalence has been reported from Beirut and Mount Lebanon among the dog owners [15].

Mean annual incidence rate of surgically treated CE cases in the current study has been determined as 1.23/100,000 inhabitants which is comparable with the incidence in other Middle Eastern countries such as Israel (2.7 ± 1.2 /100,000 inhabitants) [29] but higher than Iran (0.74/100,000 inhabitants) [30]. An incidence of 0.87–6.6 per 100,000 inhabitants has been reported for CE in Turkey [31]. Similarly, higher annual incidence rates were found in Jordan (2.3/100,000 inhabitants [32]) and Sardinia where the mean annual regional discharge rate for patients hospitalized for symptoms correlated to CE was 9.3/100,000 inhabitants [33] and 6.62/100,000 inhabitants [35]. Furthermore, the number of CE cases obtained in current study was higher than those reported by the Lebanese Ministry of Health showing that CE disease is under-reported by healthcare professionals and the general population, hence the need for applying effective strategies to strengthen health informatics and to improve data quality. The disease is disseminating in Mediterranean countries, however, the exact incidence and prevalence of CE in humans and animals remains unknown [1].

Based on surgically confirmed CE cases in this study, the age group of 30–39 years remained most vulnerable to the infection showing highest prevalence among all age cohorts; people in this age group are probably the most active in livestock rearing [33]. It was also observed that older age groups (40–49 and 50–59 years) had higher CE prevalence compared to younger ones which could indicate chronic but asymptomatic infections. Contrastingly, a study on human CE in Iraq [35] identified the highest prevalence for 21–30 years age group. Analysis of gender-based data revealed that females were more prone to CE infection than males, a finding that has been previously observed in Lebanon [36] and other Middle eastern countries like Iran [37, 38] and Iraq [35]. In contrast, higher male infection rates than female were reported for Sardinia [34]. This can be explained by the lifestyle of women, who are more likely to be in direct contact with a source of infection, since they tend to have main role in domestic activities, including food preparation and caring for the family dog [39, 40].

Regarding cysts localization, the current results showed that the liver was the most commonly affected organ, followed by the lungs, in line with a previous Lebanese study [15] and international literature [41, 42]. Single cases of rare sites of occurrence of the hydatid cyst like in the adrenal gland, brain, breast, intramuscular, myocardium, ovaries, pancreas, peri-bladder, spine, and subcutaneous were also observed which could be explained by the dissemination of cysts through lymphatic channels [43].

Molecular genotyping analysis of the partial *cox1* gene revealed the presence of the species *E. granulosus s.s.* (G1–G3 complex) with sheep strain having the predominant involvement in human CE in Lebanon, as identified in most Asiatic human populations [44, 45]. The *E. granulosus s.s.* nucleotide sequences obtained from humans in current study were similar to those identified from the goats and sheep in Lebanon [17] reflecting genetic similarity and efficient circulation of the *E. granulosus s.s.* genotypes among the intermediate hosts across different areas of the country. Five nucleotide sequences identified from the cysts localized in lungs and livers of patients were similar to the second most common haplotype identified in Iran, Turkey, and Iraq [46]. The genealogical relationships between the haplotypes were evident from the shared haplotypes between different *E. granulosus s.s.* populations. The haplotype EG4 identified in this study is considered to be a founder haplotype and is also reported from other regions like Iran and Jordan [47], Tunisia [48], and Sardinia [49]. The second most common haplotype (EG15) is reportedly present in Iran [47], Tunisia [48] and Iraq [46]. Similarity in genetic structures among different geographical regions reflects common evolutionary scenario whereby the founder haplotype seemed to have spread across continents through anthropogenic movement of the intermediate hosts from the cradle (Middle East) of this parasite [46].

Population genetics analysis further revealed the absence of genetic differentiation and high degree of gene flow among these countries as indicated by very low F_st_ values. Moreover, high haplotype diversity, low nucleotide diversity and highly negative bias from neutrality suggested population expansion and occurrence of single nucleotide substitutions as identified from various parts of the world [47, 48]. Little genetic differentiation among the *E. granulosus s.s.* isolates originating from different intermediate hosts indicates the presence of efficient transmission routes of this species to humans across different regions and among various intermediate hosts [50]. Likewise, the presence of *E. canadensis* (G7) among goats in Lebanon was recorded previously [17] and it is quite likely that this genotype could be involved in the human CE if more human isolates are characterized as it is the second most infective genotype to humans [13]. Epidemiological situation of different *E. granulosus s.l.* genotypes implicated in human CE in Lebanon may be greatly clarified with the addition of more data, provided that the concerned authorities ensure proper record maintenance and documentation.

Retrospective studies have certain limitations. A selection bias is possible since the samples were recruited from selected anatomy and pathology laboratories. The machines used for analysis differed between hospitals, which might predispose us to information and validity biases. Only CE patients who underwent surgical removal of hydatid cysts were included. For this reason, the data presented are probably underestimating the incidence of CE in Lebanon. Data concerning morbidity and mortality associated with CE, mean hospital stay and follow-up time were not available. Future studies including factors associated with CE spread, including slaughterhouses without supervision, illegitimate slaughtering, little public and farmers’ mindfulness of the hydatid disease, and the big number of homeless dogs, need to be conducted to correctly estimate the status of CE in Lebanon.

## Conclusion

This study added information about the *status quo* of CE rate in Lebanon and its distribution across the different regions of the country. As a whole, this work clearly showed that CE represents a serious health problem in Lebanon and its extent is still underestimated due to under-reporting and weak maintenance of data by concerned officials and public institutions. Rise in incidence rates of surgery, however, suggested the perpetuation of the disease and involvement of multiple risk factors which are still to be defined for the Lebanese population. Limited knowledge on disease and poor surveillance may impede the efforts of disease control as the exact burden of disease is unknown. In view of the important impact of such disease in terms of public health and economy, more efforts are needed to be taken by the local authorities to establish efficient control programs.
